# Influence of Conditions of Pd/SnO_2_ Nanomaterial Formation on Properties of Hydrogen Sensors

**DOI:** 10.1186/s11671-017-2152-3

**Published:** 2017-06-02

**Authors:** E. V. Sokovykh, L. P. Oleksenko, N. P. Maksymovych, I. P. Matushko

**Affiliations:** National Taras Shevchenko University of Kyiv, 62a Volodymyrska str, Kyiv, 01601 Ukraine

**Keywords:** Nanomaterial Pd/SnO_2_, Sensor, Hydrogen, Sol-gel method

## Abstract

Metal oxide sensors were created using nanosized tin dioxide obtained by a sol-gel method. Gas-sensitive layers of the sensors were impregnated with PdCl_2_ solutions of different concentrations to increase sensitivities of the proposed sensors. Influence of different temperature conditions of the sensor formation on the sensor properties was studied. It was found that decreasing duration of high-temperature sensor treatment prevents enlargement of particles of the gas-sensitive materials. It was shown that the sensors based on materials with smaller particle sizes showed higher sensor responses to 40 ppm H_2_. Obtained results were explained in terms of substantial influence of length of the common boundaries between the material particles of tin dioxide and palladium on the gas-sensitive properties of the sensors. The obtained sensors had possessed a fast response and recovery time and demonstrated stable characteristics during their long-term operation.

**Electronic supplementary material:**

The online version of this article (doi:10.1186/s11671-017-2152-3) contains supplementary material, which is available to authorized users.

## Background

Nowadays, hydrogen is widely used for chemical synthesis in industry and as environmentally friendly energy source [1–3]. Hydrogen is an explosive gas, and therefore, control of H_2_ content in areas of its using, transportation, and storage is needed. Gas analysis devices based on metal oxide sensors can be promising to realize such control [4–6].

It is well known that nanosized materials have some unique physicochemical properties, i.e., optoelectronic [7], magnetic [8], and catalytic [9]. SnO_2_ is a perspective material to create the metal oxide sensors due to its chemical inertness, thermal stability, and ability to chemisorb oxygen. That is why nanomaterials based on tin dioxide are very interesting to study as gas-sensitive layers of the sensors to measure H_2_ in air. Increasing the sensor responses to hydrogen can be achieved by addition in the gas-sensitive layer of the sensors of catalytic active additives including Pd which is one of the most active catalysts in a reaction of hydrogen oxidation [6, 10].

Composition of the sensor material, method of its preparation, and conditions of the material formation can influence on the particle size [11–13] and thus on gas-sensitive properties of the material.

Morphology of the material of the sensor-sensitive layer including its size of particles and their distribution has great importance to create highly efficient metal oxide sensors [14–16]. It is known that decreasing the particle size of the sensor sensitive layer material should increase the sensor response [17]. At the same time, it is known that creation of the sensors requires their high-temperature sintering. However, the high-temperature sintering leads to enlargement of the nanomaterial particles. That is why conditions of a process of the high-temperature sintering of the sensor should prevent the enlargement of the particles and provide simultaneously both mechanical strength of the sensors and their conductivities through formation of contacts between the nanoparticles of the material of the gas-sensitive layer [18].

Optimal temperature of the sensor sintering which should satisfy the conditions listed above can be achieved by required temperature values and time duration of definite stages of the high-temperature sintering of the sensors. The conditions of formation of the sensor nanomaterial should also provide full completion of crystallization and stabilization of its nanoparticles.

The aim of this work is to study the influence of conditions of formation of Pd/SnO_2_ nanomaterials with different palladium content on properties of semiconductor sensors to hydrogen.

### Methods

#### Synthesis of Nanosized Tin Dioxide

Synthesis of nanosized SnO_2_ materials was carried out by a sol-gel method. The sample of SnCl_4_·5H_2_O (*m* = 1.5 g) was dissolved in 15 ml of ethylene glycol. The obtained solution was evaporated at 110–120 °C. After evaporation of ethylene glycol, a dark brown gel was formed. The resulting gel was dried at 150 °C to form a xerogel. The xerogel was grinded up and placed on a ceramic plate. To obtain nanosized SnO_2_, thermal decomposition of the xerogel was carried out in air using a high-temperature furnace Gero (Germany). Nanosized SnO_2_, carboxymethyl cellulose, and PdCl_2_ were used to obtain the gas-sensitive materials.

#### Preparation of Adsorption-Semiconductor Sensors

**Fig. 1:**
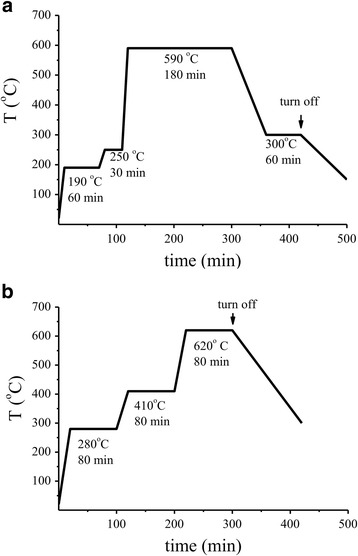
Schemes of temperature heating of the sensors based on SnO_2_. **a** Mode 1. **b** Mode 2

Adsorption-semiconductor sensors were prepared by deposition of a paste of the gas-sensitive material on the sensor ceramic plate which had measuring electrical contacts and a heater [19]. The paste was prepared by mixing the synthesized SnO_2_ nanomaterial and aqueous solution (3 wt%) of carboxymethylcellulose. A definite volume of the paste (3 μL) was placed on the sensor ceramic plate using Hamilton’s syringe 85 RN SYR (5 μL) to provide the same thickness of the sensor layer. According to SEM data, thickness of the layer of the sensor was about 70 μm (Additional file 1: Figure S1, Supporting Information Section). The sensors were dried at 90 °C during 1 h in air. Introduction of palladium to the gas-sensitive layers of the sensors was carried out by impregnating them with palladium chloride solution of certain concentrations (CPdCl_2_ = 0.05 × 10^−2^–0.15 M). After impregnation, the sensors were dried and sintered in a high-temperature furnace using two different temperature modes which included stepwise heating of the sensors (Fig. 1a, b). The sensors and gas-sensitive materials obtained by temperature heating modes 1 or 2 were named S_1_ or S_2_ respectively.

#### Methods of Measurement

To measure a value of the sensor signal, the sensors were placed into chambers and connected to a special electric stand [20]. Measuring was carried out using analyzed gas flow with a rate of 400 ml/min. Required sensor temperature was ensured by a definite value of voltage on the sensor heater. Measurement of the sensor temperature was carried using a pyrometer Optris Laser Sight (Optris, Germany). The sensors were stabilized by aging at 400 °C during 1 week in air with periodical treatment of the sensors by the hydrogen-air mixture with 1000 ppm H_2_ before measuring the gas-sensitive properties.

Ratio of a value of the electrical resistance of the sensor in air (*R*_0_) to a value of its electrical resistance in the presence of 40 ppm H_2_ (*R*_H2_) was chosen as a measure of the sensor response. The sensor response time (*t*_0.9_) was estimated as a time required for the sensor to reach 90% of an equilibrium signal value when air is replacing by an analyzed gas. The recovery time (*τ*_0.1_) was estimated as a time required for the sensor to return to 10% above the initial signal in air when the analyzed gas is replacing by air.

The characteristics of the sensors were studied using hydrogen-air mixtures with various concentration of H_2_. Mixtures of air with H_2_, CO, CH_4_, and H_2_ and CO or H_2_ and CH_4_ were used to estimate selectivity of the obtained sensors. All analyzed gas mixtures were prepared and tested in Ukrainian Centre of Certification and Metrology.

Stabilities of the responses to 40 ppm of H_2_ for the sensors S_2_ (S-67 and S-69) during 6 months of their operation were studied.

Determination of the specific sensor material surface was carried out by Brunauer-Emmett-Teller (BET) method.

Content of palladium in the sensor materials was determined by an atomic absorption method using a spectrophotometer AAS1N Carl Zeiss (Jena, Germany) with a flaming atomizer. Atomization of palladium was performed in acetylene-air flame (2350 °C).

Study of phase composition was performed using a diffractometer Bruker D & Advance (radiation Cu*Kα*). Identification of sample phase was carried out by comparison of obtained results and published crystallographic data.

Study of morphology of the sensor materials by TEM method was performed using a transmission electron microscope SELMI PEM-125 K with an accelerating voltage of 100 kV. The particle size analysis based on TEM images was carried out using the Kappa Image Base program. To obtain information on the particle size distribution for the obtained nanomaterials, about 300 particles in TEM image were taken into account.

The samples of the obtained nanomaterials were studied by FESEM method using a field emission scanning electron microscope JEOL JSM-6700F (JEOL Ltd., Japan) and HRTEM method using a transmission electron microscope JEM-2100F (JEOL Ltd., Japan).

Thickness of the sensor layer was estimated using a scanning electron microscope JEOL JSM-6060LA (JEOL Ltd., Japan) with a working voltage of 30 kV.

## Results and Discussion

The synthesized nanomaterials based on SnO_2_ [21] with an average particle size 8 nm were used to create the sensors and study influence of different temperature heating conditions of the sensor preparation on the gas-sensitive properties.

**Fig. 2:**
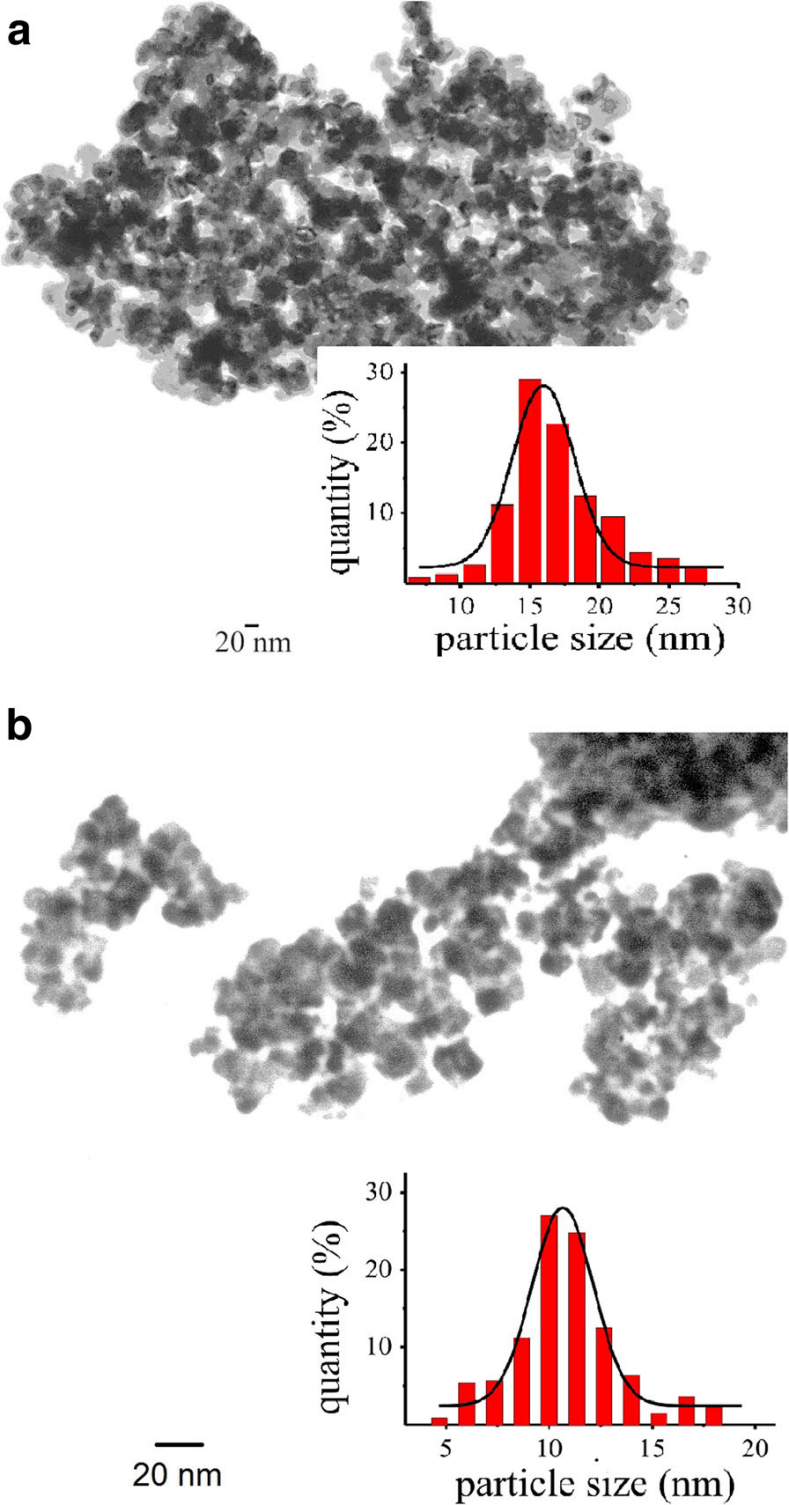
TEM images of the gas-sensitive nanomaterials **a** S_1_ and **b** S_2_

It was found earlier [19, 22, 23] that formation of the gas-sensitive layer of the sensors which had been prepared using temperature heating mode 1 with final temperature in a range of 590–620 °C during 180 min had been led to form the particles with sizes from 5 to 30 nm (an average size of 17 nm). TEM image of the sensor nanomaterial S_1_ obtained using temperature heating mode 1 with a final temperature of 590 °C is presented in Fig. 2a. The response (*R*_0_/*R*_H2_) of the sensor based on the material was equal to 6.7. Increasing the sensor response can be provided by using the materials with smaller particle sizes. Such particles can be possibly obtained using temperature heating mode with less duration at the final temperature of the sensor sintering. It was found that decreasing the duration of the sensor heating from 180 to 80 min at the final temperature of the heating mode 1 (590 °C) had led to form very small values of the sensor responses to 40 ppm H_2_ (*R*_0_/*R*_H2_ ~ 2) and high values of the electrical resistances sensors in air (>500 MOhm) for the most of the created sensors. These conditions of the sensor sintering did not probably lead to formation of sufficient quantity of the contacts between particles of the material to allow passage of electric current through the sensor.

To provide both formation of the sensor conductivity and its mechanical strength, the duration of the sensor heating was reduced up to 80 min with simultaneous increasing of the final temperature of the sensor sintering to 620 °C. Furthermore, the duration of the sensor heating in this temperature mode was increased to 80 min in low-temperature regions of the sintering, namely, at 280 and 410 °C, that corresponded to the temperatures of CMC and palladium chloride decomposition [24–26]. These changes in the low-temperature regions of the sensor formation are caused by necessity of formation of a larger number of the contacts in the sensor material. The increasing of the particle size of the material in the low-temperature regions should not certainly be so intense as it should be at 620 °C. Scheme of more soft temperature heating mode 2 of the sensor sintering is presented in Fig. 1b.

Analysis of TEM micrographs of the obtained sensor materials S_2_ (Fig. 2b) showed that these materials include particles which were smaller than particles of the sensor material S_1_ (Fig. 2a): an average particle size of tin dioxide for both studied temperature heating modes 1 and 2 was 17 and 11 nm respectively. Such decreasing of the particle size of the sensor material S_2_ contributed to an increase of value of tin dioxide specific surface to 47 m^2^/g instead of 39 m^2^/g which was found for the sensor material S_1_.

It was shown that palladium content in the Pd/SnO_2_ nanomaterials obtained by impregnation of the nanosized SnO_2_ by solutions of PdCl_2_ increases when concentration of palladium chloride increases too. In particular, when concentration of PdCl_2_ solution was changed from 0.05 mol/L to 15 × 10^−2^ mol/L, the content of palladium additives in the nanomaterials was changed from 0.001 to 0.193 wt%.

According to XRD data, unmodified tin dioxide and Pd/SnO_2_ nanomaterials with different palladium content obtained in both temperature modes have cassiterite structure with identical lattice parameters *a* = 0.4738 nm, *b* = *c* = 0.3187 nm [21].

**Fig. 3:**
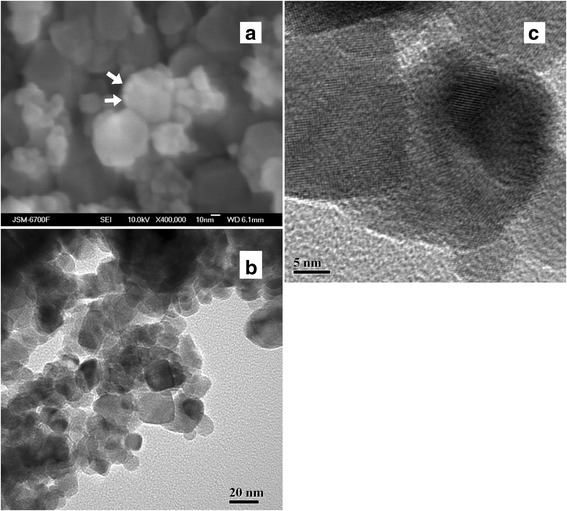
**a** FESEM and **b**, **c** HRTEM images of the sensor Pd/SnO_2_ nanomaterial

FESEM image of the obtained sensor material (Fig. 3a) demonstrates the grains of SnO_2_ nanomaterial and Pd particles (shown by arrows in Fig. 3a). Clear boundaries between the particles of the sensor nanomaterial can be seen on the HRTEM images (Fig. 3b, c).

**Fig. 4:**
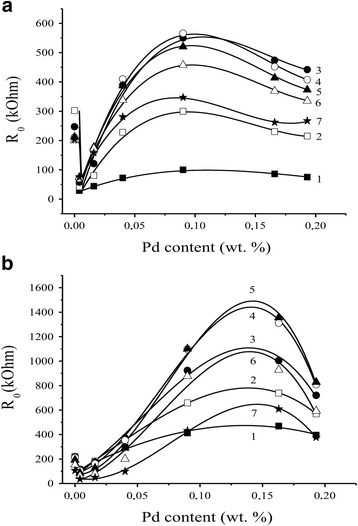
Dependence of *R*_0_ values of the sensors **a** S_1_ and **b** S_2_ based on Pd/SnO_2_ on the palladium content at different temperatures of the sensors: *1* 410 °C, *2* 382 °C, *3* 355 °C, *4* 327 °C, *5* 295 °C, *6* 261 °C, and *7* 225 °C

It was shown (Fig. 4a, b) that the dependence of the electrical resistance values in air of the Pd-containing sensors on palladium content at different sensor temperatures has a complicated character with minimum at low palladium contents and wide maximum at much higher Pd contents for both different temperature heating modes of the sensors.

To explain the obtained results, it should be noted that values of the resistance *R*_0_ and sensor response with addition of metals (or oxides) in material of the gas-sensitive layer were provided by formation of common boundaries between particles of the active additives and tin dioxide [19, 27, 28]. When the sensor is heated in air, these boundaries take part in chemisorption of oxygen with localization of electrons from conductivity band of semiconductor. Such chemisorption influences on the values of the electrical resistance of the sensor. In the presence of an analyzed gas, a heterogeneous catalytic reaction oxidation of the gas by chemisorbed oxygen is running on the surface of the semiconductor. The electrons localized at chemisorbed oxygen return to the conductivity band of the semiconductor, and the decrease in the electrical resistance of the sensor is performed. In this case, the stationary oxygen quantity on the sensor surface that occurs as a result of a dynamic equilibrium state of the oxidation reaction will determine the resistance value of the sensor. A change of the value of the sensor resistance when air is replaced by the analyzed gas determines the value of the sensor response. Under identical conditions (the same gas of the definite concentration and the same temperature of the sensor), the value of the electrical resistance of the sensor in air and its change in the presence of the analyzed gas (sensor response) will depend on the length of the boundary between palladium and tin dioxide particles.The palladium content in the sensor material will affect the value of the length of the boundary and thus will determine the properties of the sensor.

As can be seen from Fig. 4a, b, introduction of palladium (up to 0.05% Pd) affects the sensor value *R*_0_ in the same manner independently of the temperature heating mode of the sensor sintering. The observed initial reduction of the value of the sensor electrical resistance may be occurred as a result of existence of metallic palladium which is formed on the sensor surface according to the obtained XPS data [19]. Further increase of palladium content leads to a slight increase in the values of the resistances of the sensors S_1_ and S_2_ due to the low oxygen chemisorption at the boundary of a very small length between SnO_2_ and palladium particles. It should be noted that similar values of the resistances of the sensors S_1_ and S_2_ in the range of such low palladium contents indicate no significant influence of palladium on the properties of the sensors which are determined by own properties of tin dioxide in these conditions. It was found that the value of electrical resistance of SnO_2_ did not practically depend on the sintering temperature of the sensor in the temperature range of 590–620 °C as it was found in [19, 21–23].

Change of the temperature heating mode of creation of the sensors S_1_ and S_2_ affects the value of their resistance significantly when palladium content is increased (>0.05% Pd) (Fig. 4a, b). Indeed, the resistance for the sensors S_2_ have much greater values than those for the sensors S_1_ in conditions of the same palladium contents in the concentration range of 0.05–0.2% Pd. This is in agreement with the assumption about a stabilizing role of palladium [29] which prevents the enlargement of the nanomaterial particles, and the soft temperature heating mode 2 of the sensor sintering contributes to this process. The length of the boundaries between particles of palladium and tin dioxide under these soft temperature conditions will be longer for the material S_2_, and therefore, due to a large quantity of oxygen chemisorbed on the boundaries, the values of resistance for the sensors S_2_ should be greater than those for the sensors S_1_. This is confirmed in an experiment (Fig. 4a, b). Formation of the smaller particles for the Pd-containing nanomaterials obtained in the soft temperature conditions of the heating mode 2 was also confirmed by TEM method (Fig. 2b).

Finally, at very high palladium contents, process of Pd particle aggregation can start and it will decrease the length of the common boundaries resulting in decrease in the electrical resistance values of the sensors (Fig. 4b).

**Fig. 5:**
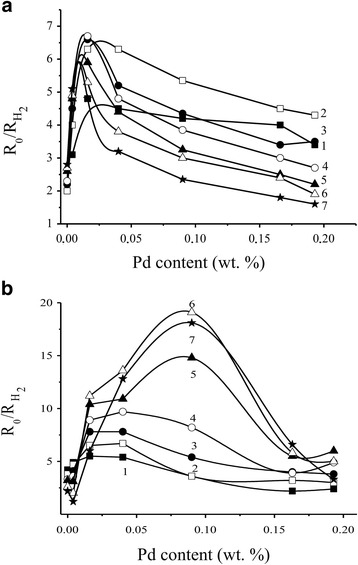
Dependence of the sensor response to 40 ppm H_2_ for the sensors **a** S_1_ and **b** S_2_ based on Pd/SnO_2_ on the palladium content at different temperatures of the sensors: *1* 410 °C, *2* 382 °C, *3* 355 °C, *4* 327 °C, *5* 295 °C, *6* 261 °C, and *7* 225 °C

In general, a change of the sensor responses to hydrogen is correlated with a change in their electrical resistance (Figs. 4a, b and 5a, b): an increase in the values of the electrical resistance of the sensors leads to an increase in the values of their sensor responses to H_2_. The responses of the sensors S_2_ to 40 ppm of hydrogen are higher than the responses of the sensors S_1_ (Fig. 5). As it can be seen (Fig. 5), decreasing the sensor response to H_2_ is observed for the highest contents of Pd additives in comparison with the sensor S_2_. It can probably be due to aggregation of the palladium clusters which cover the semiconductor surface to a great extent, and the tin dioxide surface becomes unavailable for hydrogen. That is why decreasing the sensor response is observed in experiment.

It was found that positions of maximum values of the sensor electrical resistances (Fig. 4a, b) and the sensor responses (Fig. 5a, b) for the sensor S_2_ compared to the sensor S_1_ are shifted to a region of the higher palladium contents. It can be a result of existence of relatively larger palladium content on the sensor surface in a non-aggregated state for the material SnO_2_ with smaller size of their particles. Such state of the material will promote increase of the electrical resistance value of the sensor in air and the sensor response to hydrogen.

**Fig. 6:**
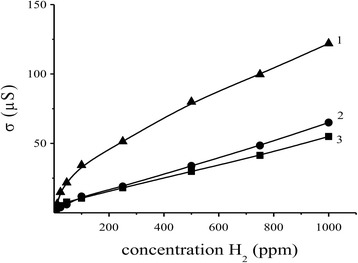
Dependence of the conductivity of the sensor S_2_ based on 0.09% Pd/SnO_2_ nanomaterial on the hydrogen concentration at different sensor temperatures: *1* 261 °C, *2* 327 °C, *3* 382 °C

For the most sensitive sensor S_2_ based on 0.09% Pd/SnO_2_ nanomaterial, other sensor properties were studied. It was found that this sensor is sensitive to hydrogen in a wide range of its concentrations at the different sensor operation temperatures (Fig. 6). A dependence of conductivity of the sensors on H_2_ concentration is practically linear in a tested range of H_2_ concentration (2–1000 ppm H_2_) at the different sensor temperatures (327 and 382 °C) (Fig. 6). Non-linearity of sensor conductivity in the wide range of H_2_ concentration at 261 °C is probably associated with various energy bond of chemisorbed oxygen on the sensor surface. It was found that a detection limit of H_2_ measurement for the most sensitive sensor is equal to 2 ppm in air. A change of the sensor conductivity which reaches to 44–52% for such low hydrogen concentration depends on the sensor temperature. It should be noted that the response to 2 ppm H_2_ (*R*_0_/*R*_H2_ = 2.1 at 261 °C) for the created sensor is higher than a response to the same H_2_ concentration (*R*_0_/*R*_H2_ = 1.3 at 265 °C) for the sensor based on nanosized SnO_2_ studied in [30].

**Fig. 7:**
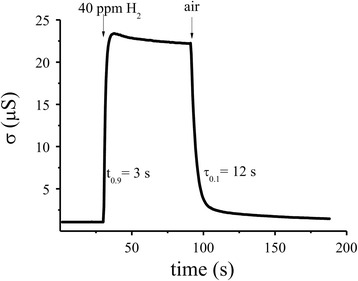
Change in the conductivity versus time for the optimal Pd-doped sensor (0.09% Pd/SnO_2_) at the sensor temperature 261 °C

It was shown that the sensor based on S_2_ material (0.09% Pd/SnO_2_) possess a fast response (*t*_0,9_ = 3 s) and recovery (*τ*_0.1_ = 12 s) time at 261 °C (Fig. 7). It should be noted that created sensors have also a high sensor response (*R*_0_/*R*_H2_ = 19.5) to microconcentration (40 ppm H_2_) of hydrogen. It is much better in comparison with corresponding characteristics of the sensor based on Pd/SnO_2_ nanomaterial studied in [31] where the sensor response to 50 ppm H_2_ is equal to *R*_0_/*R*_H2_ = 15.9 and the response and recovery time are equal to *t*_0,9_ = 120 s and *τ*_0.1_ = 15 min.

**Fig. 8:**
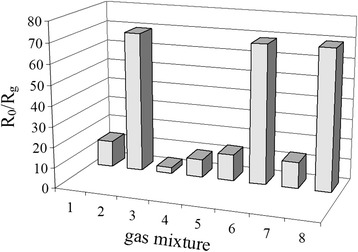
Response of the sensor S_2_ containing 0.09 wt% Pd (*T* = 261 °C) upon exposure to analyzed gas mixture of air with *1* 20 ppm H_2_, *2* 500 ppm H_2_, *3* 500 ppm CH_4_, *4* 500 ppm CO, *5* 20 ppm H_2_ + 500 ppm CH_4_, *6* 500 ppm H_2_ + 500 ppm CH_4_, *7* 20 ppm H_2_ + 500 ppm CO, and *8* 500 ppm H_2_ + 500 ppm CO

The results in the study of selectivity to H_2_ for the sensors S_2_ containing 0.09 wt% Pd (*T* = 261 °C) in the presence of CO and CH_4_ are shown in Fig. 8. Comparison of the sensor response to H_2_, CH_4_, or CO of the same concentration (500 ppm) shows that the sensor response to H_2_ is much higher than for CH_4_ or CO. That is why the presence of CH_4_ or CO of 500 ppm concentration in the analyzed gas mixture with 500 ppm of H_2_ does not practically influence on hydrogen measurement (Fig. 8). Such influence is also absent for measurement of microconcentration of H_2_ (20 ppm) in the case of its mixture with 500 ppm of CH_4_ or CO. Such behavior of the sensors can be explained by different values of the optimal sensor temperature needed to provide the maximal value of sensor response for each of the tested gases. The optimal temperature of the sensor to measure H_2_ is much lower (261 °C) than that for CH_4_ (382 °C) and CO (327 °C). Low sensor temperature to measure H_2_ is explained by a higher activity of H_2_ compared with CH_4_ and CO activities in oxidation reaction on the sensor surface. A practical absence of interference from CH_4_ and CO for the sensor response to H_2_ (Fig. 8) in the studied conditions can be also explained by a predominant hydrogen oxidation reaction running on the surface due to higher reactivity of H_2_ compared to CO and CH_4_.

**Fig. 9:**
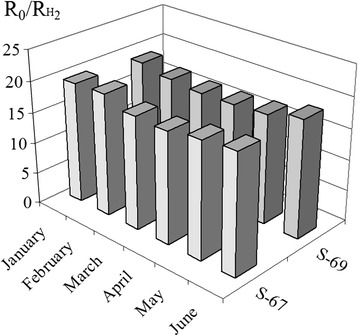
Response to 40 ppm H_2_ of the sensors S_2_ (S-67 and S-69) based on 0.09% Pd/SnO_2_ nanomaterial during 6 months of their operation at the sensor temperature 261 °C

Stability of the sensor response of long-term operation for two sensors S_2_ based on 0.09% Pd/SnO_2_ nanomaterial during 6 months was studied. It was found that the sensors S_2_ did not lose their sensor responses and did not have any directed drift of the sensor response after 6 months of the sensor operation (Fig. 9). This result shows a possibility to apply the created sensors in practice.

## Conclusions

Change of conditions of high-temperature treatment of the sensors based on Pd/SnO_2_ led to form smaller particles of nanomaterial of the gas-sensitive layer of the sensor that allowed to reach a significant value of the sensor response (*R*_0_/*R*_H2_ = 19.5) to microconcentration of hydrogen (40 ppm) at the sensor temperature 261 °C. The created sensors can measure hydrogen in a wide range of its concentration (2–1089 ppm H_2_), have a low limit of H_2_ detection, and demonstrate a fast response and recovery time. The created sensors are stable during their long-term operation.

## Additional file

Additional file 1: Fig. S1. SEM image of the gas sensitive layer deposited on the sensor plate. (JPEG 560 kb)
