# Supplementary figures and images for: Influence of Conditions of Pd/SnO2 Nanomaterial Formation on Properties of Hydrogen Sensors
Source: Nanoscale Res Lett. 2017 Jun 2;12:383. doi: 10.1186/s11671-017-2152-3 (PMC5457378; doi:10.1186/s11671-017-2152-3)

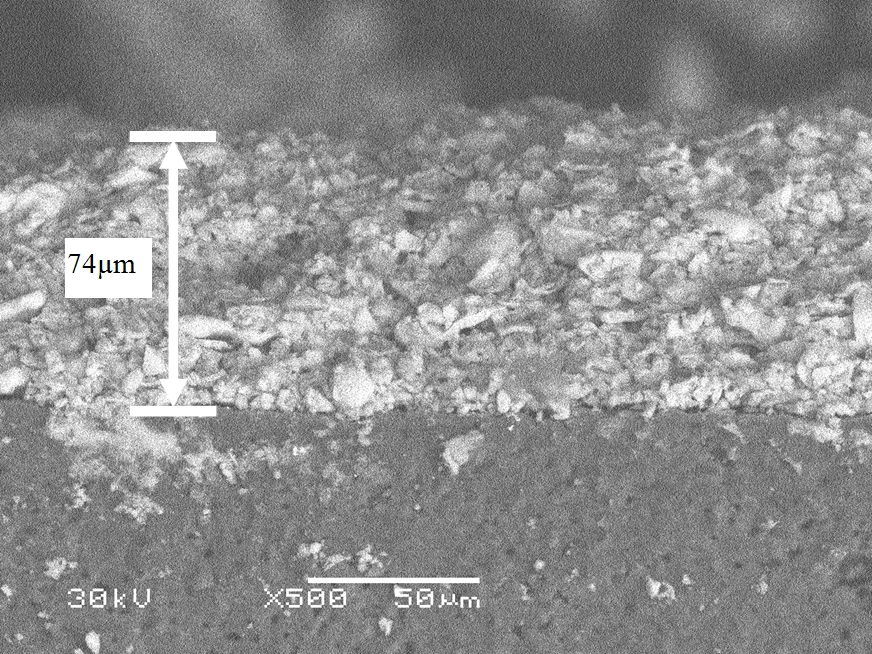

Supplement: Additional file 1: Fig. S1. — SEM image of the gas sensitive layer deposited on the sensor plate. (JPEG 560 kb) [file 11671_2017_2152_MOESM1_ESM.jpeg]
